# Processing Wastewaters from Spanish-Style cv. Chalkidiki Green Olives: A Potential Source of *Enterococcus casseliflavus* and Hydroxytyrosol

**DOI:** 10.3390/microorganisms8091274

**Published:** 2020-08-21

**Authors:** Eugenia Papadaki, George Botsaris, Eleftheria Athanasiadi, Fani Th. Mantzouridou

**Affiliations:** 1 Laboratory of Food Chemistry and Technology, Department of Chemistry, Faculty of Sciences, Aristotle University of Thessaloniki, 54124 Thessaloniki, Greece; epapadaki@chem.auth.gr (E.P.); aelefther@chem.auth.gr (E.A.); 2 Department of Agricultural Sciences, Biotechnology and Food Science, Faculty of Geotechnical Sciences and Environmental Management, Cyprus University of Technology, Limassol 50329, Cyprus; george.botsaris@cut.ac.cy

**Keywords:** table olive wastewaters, lactic acid bacteria, *Enterococcus casseliflavus*, *Bacillus amyloliquefaciens* subsp. *plantarum*, 16S rRNA gene sequencing, bioaugmentation, oleuropein hydrolysis, Hydroxytyrosol

## Abstract

The purpose of this study was to examine the isolation of indigenous lactic acid bacteria (LAB) with functional properties from Spanish-style cv. Chalkidiki green olive processing wastewaters (GOW). Predominant indigenous LAB could serve as bioaugmentation agents/starter culture for table olives production and protected designation of origin specification. Spontaneous fermentation of fresh GOW over different temperatures (15 °C to 50 °C) and pH values (3.5 to 11.5) for 30 d enabled the isolation/molecular identification of the lactic acid bacterium *Enterococcus casseliflavus* and the plant-associated bacterium *Bacillus amyloliquefaciens* subsp. *plantarum*. *E. casseliflavus* was found to reduce chemical oxygen demand by 72%. Its resistance to extreme pH values, salinity, and temperature was successfully modeled and the minimum inhibitory concentration of oleuropein against the bacterial growth was determined (0.9 g/L). Furthermore, hydroxytyrosol content was doubled (up to 553 mg/L) after GOW spontaneous fermentation under acidic conditions at 15 °C to 30 °C for 120 d, creating an additional source of input. These results highlight the significance and potential of *E. casseliflavus* in Spanish-style cv. Chalkidiki green olive processing.

## 1. Introduction

Table olives constitute a significant commodity traded in the global food market. Notably, the world production of table olives has nearly doubled during the period 2000–2018, reaching about 3 million tonnes [1]. This increase coincided with the generation of large volumes of polluting wastewaters from the various stages of table olive processing. For Spanish-style green olive processing, which is one of the most commonly used methods for table olive production, an annual generation of 5 to 6 million m^3^ of polluting wastewaters was estimated over the period 2013–2018 [1,2]. Among the resulting wastewaters, lye and washing water effluents (Spanish-style green olive processing wastewaters, GOW) account for 75% of the total wastewater volume. Considering that these effluents are strongly alkaline, contain highly phytotoxic pollutants (mainly phenolic compounds and sodium ions), and are generated within a short time (~1 month) and space, their management is an emerging need [2,3].

Despite the very different physicochemical characteristics of GOW, there are no specific legal regulations towards their efficient management. Directive (EU) 2018/851, an amendment to the waste framework directive 2008/98/EC, encourages the respective entities to develop processes for the simultaneous wastewater detoxification and valorization in the context of the modern trend towards circular economy [4]. The most common practice is their disposal in evaporation ponds, causing serious damages to the natural habitat (e.g., emission of malodorous gases, contamination of groundwater/deep soil). The development of sustainable remediation processes is a tough challenge and bioaugmentation via the use of exogenous fungi (white-rot fungi, *Aspergillus niger*, *Geotrichum candidum*, *Trichoderma harzianum*), microalgae (*Nannochloropsis gaditana*), or activated sludge has emerged as a feasible, green, and cost-effective approach. However, recent findings highlight the limited adaptability of these microbial cells to GOW and their limited ability to degrade the toxic organic constituents (i.e., phenolic compounds) [2,5,6]. *A. niger* has been recognized as metabolically superior against the other tested microorganisms in terms of phenol degradation and phytotoxicity reduction [3]. With the same target in mind, the simultaneous wastewater detoxification and valorization for the production of specialty chemicals (e.g., methane, citric acid, lactic acid, hydroxytyrosol) has been proposed to create an additional source of input alongside the reduction of the disposal costs, in the context of the circular economy [2,7].

The exploitation of indigenous microbial communities as bioaugmentation agents in waste is anticipated to display adequate adaptability and enzymatic activities for in situ breakdown and mineralization of a wide range of pollutants, promoting the development of more effective biorefinery processes [8]. However, relevant research studies in lye and washing waters from Spanish-style green olive processing are incomplete and non-systematic. Specifically, the existing knowledge in the indigenous microflora of table olive processing wastewaters is limited to the isolation of *Bacillus* sp. WW3-SN6 and *Alkalibacterium olivoapovliticus* from fresh washing waters (initial pH value 11–13) [9,10]. In the abovementioned studies, the bacterial isolates were characterized as alkaliphilic, halotolerant and psychrotolerant. Nevertheless, none of the available studies tested the potential of the isolates as bioaugmentation agents for table olive processing wastewaters.

Unlike GOW, several studies have focused on the isolation of indigenous cultivable lactic acid bacteria (LAB) from the fermentation brine of Spanish-style green olive processing, such as *Lactobacillus* spp., *Enterococcus* spp., and *Leuconostoc* spp., and their use as indigenous starter cultures (single or mixed), e.g. [11,12]. Research towards this direction is important considering that the adaptation of indigenous starter cultures in the process environment is typically better than that of the exogenous ones, improving the fermentation control. More importantly, the indigenous LAB are usually the only ones allowed for protected designation of origin (PDO) table olive production since, in this way, the product remains connected with its geographical origin [13]. The exploitation of multifunctional starters related to olives is anticipated to improve table olive processing by decreasing the fermentation time, contamination risk, and operation cost, as well as by enhancing the quality and ensuring the safety of the final product [14,15]. The isolation of predominant cultivable LAB in lye and washing waters, which demonstrate the ability to grow at the high initial pH values of the fermentation brine, due to the debittering stage, seems very promising and the way forward towards the development of efficient and effective starters in olive fermentation.

This study aimed to (i) screen and identify the dominant cultivable LAB involved in spontaneous fermentation of GOW under different environmental conditions (pH, temperature), (ii) assess the effectiveness of selected isolate(s) on bioaugmentation of fresh GOW, and (iii) evaluate the growth performance of the isolate(s) under the factors associated with the Spanish-style cv. Chalkidiki green olive fermentation environment (pH value, salinity, temperature, oleuropein concentration). To create an additional source of input, the evolution of hydroxytyrosol content during the spontaneous fermentation was also assessed.

## 2. Materials and Methods

### 2.1. Sampling

Olives (cv. Chalkidiki) were hand-harvested at mature greenish-yellow stage of ripening in October 2017. The olive flesh was subjected to lyophilization and stored at −20 °C until further analysis. A representative sample of fresh GOW (50 L) was obtained from a medium-sized enterprise located in Chalkidiki (Greece) in October 2016 by mixing lye and washing water effluents (ratio 1/2, *v*/*v*). The latter streams were collected after olive treatment with 2% (*w*/*v*) NaOH aqueous solution (11 h) and two washings (8 h each), respectively, from three different tanks filled with 8 tonnes of olives and 5000 L liquid.

### 2.2. Fermentation Conditions of Spanish-Style Green Olive Processing Wastewaters

The sample of GOW was split into three sub-samples (GOW-A, GOW-B and GOW-C) of 10 L. The initial pH value of GOW-A was not adjusted (11.5), while those of GOW-B and GOW-C were adjusted to 5.0 and 3.5, respectively, with HCl (37%, *w*/*w*). Each sub-sample was divided into four sets of 5 × 500 mL DURAN glass bottles, which were then hermetically sealed with a screw cap. Each set of bottles was subjected to spontaneous fermentation under static conditions (dissolved oxygen concentration <4%) in the dark at 15 °C, 30 °C, 50 °C, or room temperature for 360 d.

### 2.3. Enumeration and Isolation of Lactic Acid Bacteria

LAB enumeration was performed on Man, Rogosa, and Sharpe (MRS) agar (Lab M Limited, Heywood, UK) supplemented with 0.01% cycloheximide (JK Scientific, Pforzheim, Germany) under anaerobiosis (37 °C, 72 h). The results were expressed as colony forming units (CFU) in wastewater (CFU/mL). Representative isolates were selected (~20% of total colonies per plate) [16], purified on MRS agar and stored (20% glycerol, −80 °C).

### 2.4. Characterization of Isolates

#### 2.4.1. Phenotypic Characterization

A Gram stain kit (Liofilchem, Abruzzo, Italy) was used to differentiate the bacteria by their cell wall properties, according to the Gram staining technique. For catalase testing, a drop of 3% (*v*/*v*) H_2_O_2_ was added to each colony on a sterile glass slide and the generation of O_2_ (bubble formation) was observed. Microscopic observation of the cellular morphology was performed with a microscope (100× magnification) (Optika Srl, Ponteranica, Italy).

#### 2.4.2. Molecular Identification

An aliquot of bacterial stocks was incubated in MRS broth at 37 °C for 24–48 h to obtain a sufficient amount of cell biomass. At the end of incubation, a portion (1 mL) was transferred into an Eppendorf tube (1.5 mL), centrifuged at 14,000× *g* for 2 min and the supernatant was discarded. The procedure was performed once more and the precipitated cells were used for DNA isolation by the GenElute Mammalian Genomic DNA Purification Kit Protocol (Sigma-Aldrich, Steinheim, Germany).

DNA amplification was realized with two primers that targeted the V3 region of the 16S rRNA gene (V3f, CCTACGGGAGGCAGCAG and V3r, ATTACCGCGGCTGCTGG) (Eurofins Genomics, Ebersberg, Germany). Polymerase chain reaction (PCR) was performed on an automated DNA thermal cycler (Techne Progene, Staffordshire, UK) according to the protocol described in Muyzer et al. [17], with some minor modifications. Specifically, the cycle sequencing consisted of 10 cycles of 94 °C (5 min), followed by 10 cycles of 94 °C (1 min), 66 °C decreasing by 1 °C/cycle to 57 °C (1 min) and 72 °C (1 min). Subsequent steps included 20 cycles of 94 °C (1 min), 56 °C (1 min) and 72 °C (1 min), followed by heating at 72 °C (1 min) and then at 94 °C (1 min). The PCR products were analyzed by electrophoresis in a 2% agarose gel, stained with SYBR Safe DNA Gel Stain (Thermo Fisher Scientific, Darmstadt, Germany), and examined under UV illumination. The PCR products were then purified using the NucleoSpin^®^ PCR clean-up kit (Macherey-Nagel, Düren, Germany) according to the manufacturer’s instructions. The purified DNA was subjected to genetic analysis (ABI Prism 3130, Applied Biosystems, Foster City, CA, USA) and the identification of bacterial isolates was performed by comparing the 16S rRNA sequences of each isolate with those reported in the EzBioCloud database (https://www.ezbiocloud.net/, accessed March 2017).

### 2.5. Screening for Technological Characteristics

#### 2.5.1. Bacterial Inoculum Preparation

Inoculum of a selected bacterial isolate was prepared by transferring a loopful of cells from the stock culture to 70 mL of MRS broth (initial pH value 6.4 ± 0.2) in hydrophobic cotton-stopped 100 mL wide-mouth Erlenmeyer flasks. Cultures were incubated at 37 °C for 24 h under static conditions to a final optical density value at 630 nm (OD_630_) of 1.270 ± 0.008 (5 × 10^7^ CFU/mL).

#### 2.5.2. Bioaugmentation Ability

Hydrophobic cotton-stopped 100 mL wide-mouth Erlenmeyer flasks containing 50 mL of the sterile GOW were inoculated with 5 mL of the cell suspension (Section 2.5.1) and incubated at 37 °C for 7 d under static conditions. Control experiments using GOW without inoculation took place under the same conditions. Bacterial growth was monitored by the measurement of OD_630_ values. The flasks were withdrawn at defined time points and the treated wastewater was used for further analysis.

#### 2.5.3. Resistance to pH, Salinity and Temperature

An unblocked central composite design of the response surface methodology was used to study the effect of three factors (*X*_i_), i.e., initial pH value (*X*_1_), NaCl content (*X*_2_, % *w*/*v*), and temperature (*X*_3_, °C) on the growth of a selected isolate determined by cell density (OD_630_) (*Y*). All factors were studied at five experimental levels (Table S1). The design consisted of 20 experimental runs (Table 1), generated by Minitab software (v. 17, Product Installation CD Demo, Minitab Inc., State College, PA, USA). Six of them were conducted at the center of the design, replicated for the estimation of error. Experiments were carried out in hydrophobic cotton-stopped 100 mL wide-mouth Erlenmeyer flasks containing 70 mL of MRS broth modified by adjusting the initial pH value and NaCl content (Table 1). Each flask was inoculated with 3.5 mL of the cell suspension (Section 2.5.1) and then incubated at different temperatures for 3 d under static conditions.

The second-order polynomial model was fitted to the response, Y, giving an equation (Equation (1)) of the form:

(1) $$Y=\beta_{0}+\beta_{1}X_{1}+\beta_{2}X_{2}+\beta_{3}X_{3}+\beta_{11}X_{1}^{2}+\beta_{22}X_{2}^{2}+\beta_{33}X_{3}^{2}+\beta_{12}X_{1}X_{2}+\beta_{13}X_{1}X_{3}+\beta_{23}X_{2}X_{3}$$

where *Y* is the dependent variable; *X*_1_, *X*_2_, and *X*_3_ are the independent variables as mentioned above; and *β*_0_, *β*_1_…. *β*_23_ represented the estimated coefficients with *β*_0_ having the role of a scaling constant.

Analysis of variance was used to evaluate the quality of the fit of the model to the response by determining the coefficients of determination (*R*^2^-adj, *R*^2^-pred), the significance of each parameter through F-test (calculated *p*-value), and the lack-of-fit of the model. Coefficients with a *p*-value lower than 0.05 were considered significant. The reduced model was obtained by retaining only the significant terms and those that supported the hierarchical principle. The combination of factor values resulting in the optimum response was verified by conducting a simulation experiment in triplicate and the results were compared with model prediction outcomes.

#### 2.5.4. Resistance to Oleuropein

The minimum inhibitory concentration (MIC) of oleuropein that prevents the visible growth of a selected isolate under specified conditions was evaluated. In particular, the polar extract from olive fruit (Section 2.6.3) was filter-sterilized through a 0.22 µm polytetrafluoroethylene membrane filter, and then used as a substrate, without or after appropriate dilutions to achieve the initial oleuropein concentration in the range of 0.1 g/L to 4 g/L. Next, 1 mL of the substrate enriched with glucose (1%, *w*/*v*) and yeast extract (0.3%, *w*/*v*) (initial pH value 6.7) [18] was transferred into an Eppendorf tube (1.5 mL) and inoculated with 100 µL of the cell suspension (Section 2.5.1). The tubes were incubated at 37 °C for 3 d under static conditions. Non-inoculated substrate was also incubated under the same conditions and used as control. At the end of incubation, the enumeration of LAB (CFU/mL) was performed on MRS agar and the concentration of oleuropein in the polar fraction of the fermented substrate was determined by reversed-phase high-performance liquid chromatography (RP-HPLC) analysis (Section 2.6.3).

### 2.6. Methods

#### 2.6.1. Determination of Chemical Oxygen Demand and pH

Chemical oxygen demand (COD) (g/L) was determined by the potassium dichromate method using tube tests and an AL200 COD VARIO Set-Up (Aqualytic, Dortmund, Germany). The pH value was measured using a MP 220 pH meter (Mettler-Toledo, Greifensee, Switzerland).

#### 2.6.2. Determination of Soluble Sugar and Nitrogen Content

Total soluble sugar content (g/L), glucose (g/L), and fructose (g/L) were quantified by HPLC analysis as described elsewhere [7]. Total nitrogen content (mg/L) was assessed by the persulfate digestion method using a total nitrogen kit LCK 338 and a DR 3900 spectrophotometer (Hach Lange, Düsseldorf, Germany).

#### 2.6.3. Determination of Polar Phenolic Compound Content

The protocol used for the extraction of phenolic compounds from the olive fruit is given in Blekas et al. [19]. The methanolic extracts of 15 successive extractions were combined, the solvent was evaporated under vacuum at ~35 °C, and the dry residue was dissolved in deionized water (15 mL). Aliquots of the extract were used for the determination of the MIC of oleuropein (Section 2.5.4). The polar extracts from the wastewater samples (Section 2.2 and Section 2.5.2) and from the substrate used in the experiments for the MIC determination (Section 2.5.4) were obtained according to the liquid-liquid extraction procedure described in Papadaki et al. [3]. Total polar phenol content in the polar extracts was estimated by the Folin–Ciocalteu assay and the results were expressed as caffeic acid equivalents (mg/L). The content of individual phenolic compounds (mg/L) in the polar extracts was estimated by RP-HPLC analysis [3].

### 2.7. Statistical Analysis

All measurements and treatments were performed in triplicate. Statistical comparisons of the mean values were carried out by one-way ANOVA, followed by the Duncan’s test (*p* < 0.05 significance level) using the SPSS v. 20.0 software (SPSS Inc., Chicago, IL, USA).

## 3. Results and Discussion

### 3.1. Growth of Lactic Acid Bacteria during Spontaneous Fermentation of Spanish-Style Green Olive Processing Wastewaters

**Figure 1:**
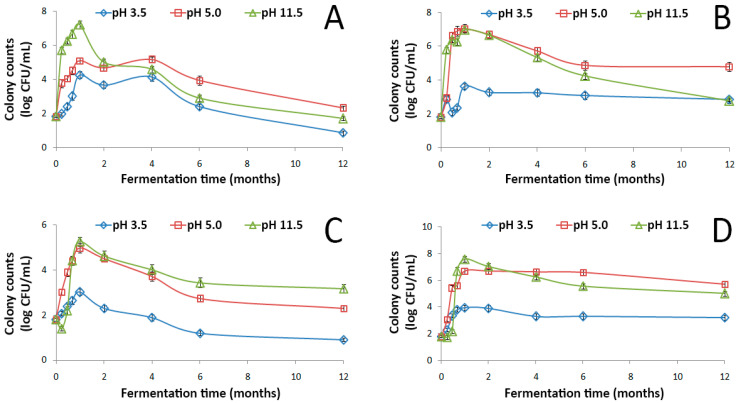
Changes in the population of LAB during the spontaneous fermentation of Spanish-style green olive processing wastewaters with an initial pH value of 3.5, 5.0 or 11.5 at 15 °C (**A**), 30 °C (**B**), 50 °C (**C**), and room temperature (**D**). Data points are mean values of 5 independent experiments × 3 measurements (*n* = 15) and error bars represent the standard deviation of the mean value.

The evolution of indigenous LAB during spontaneous fermentation of GOW at different initial pH values (3.5, 5.0, and 11.5) and temperatures (15 °C, 30 °C, 50 °C, and room temperature) throughout the year of storage are given in Figure 1.

In all tested conditions, spontaneous fermentation of GOW was feasible. The growth pattern of LAB was almost uniform during fermentation in the different treatments applied. In GOW with initial pH value uncorrected (11.5), LAB population increased gradually within ~30 d up to a maximum population of 8 log CFU/mL at room temperature and 7 log CFU/mL at 15 °C and 30 °C. LAB growth resulted in the initial pH value reduction of GOW from 11.5 to 7.6–8.7 and from 5.0 to 3.9–4.2 (Figure S1) as a result of organic acid production (mainly lactic acid) through their primary metabolism. From this maximum, the population started to decline until the end of the storage period up to a value of 5 log CFU/mL at room temperature, 3 log CFU/mL at 30 °C, and 2 log CFU/mL at 15 °C. Noticeably, LAB population in the alkaline GOW was comparable at all growth stages to that recorded in the GOW with initial pH value corrected to 5.0 at room temperature and at 30 °C but 1.5-fold higher during the first 30 d of fermentation at 15 °C (Figure 1). The latter could be attributed to the decrease of the membrane permeability at the low temperature, leading to the decline of the substrate affinity of the bacterial cells [20], along with the variation in the membrane lipid composition at different pH values. Indeed, as observed for the facultative alkaliphilic *Bacillus* sp. WW3-SN6 isolated from alkaline washing waters, the levels of phosphatidylglycerol and phosphatidylethanolamine at a pH value of 10.5 were 1.3-fold higher and 43-fold lower, respectively, than at 7.0 [9]. In both treatments, LAB population showed decreased levels (up to 5 log CFU/mL) throughout the storage period when increasing the growth temperature to 50 °C. A possible explanation is that the heat stress conditions can strongly increase the fluidity of cell membrane, rendering it unstable [21]. Beyond the fermentation temperature, LAB growth and metabolism were negatively influenced in treatment initially adjusted to 3.5 by the use of HCl. This finding ties well with that observed during the spontaneous fermentation of the washing waters from Spanish-style processing of cv. Manzanilla olives and has been correlated to the anti-LAB activity of HCl [22].

From the above results, it is clear that the LAB that survived the lye treatment of the olive fruit developed the ability to grow in the alkaline environment of GOW by maintaining the intracellular pH at a normal level [23]. In addition, alkaline stress increased the expression of genes associated with nucleotide synthesis, cell membrane properties, inorganic element transfer, and cell protection mechanisms [24]. However, the high content of polar phenolic compounds with antibacterial activity in GOW (651.6 mg/L ± 12.8 mg/L) as well as the low sugar (glucose 2.5 g/L ± 0.0 g/L, fructose 2.6 g/L ± 0.0 g/L) and nitrogen content (124.8 mg/L ± 1.5 mg/L) reflected a prolonged growth of the LAB [25].

### 3.2. Phenotypic Characterization and Molecular Identification of the Isolates

A total of 107 colonies were isolated on the selective medium MRS agar. According to the findings, there were two predominant phenotypes of the isolated colonies. More specifically, 72 colonies had white color, spherical shape, and smooth surface (Group A), while the remaining 35 had white color, irregular shape, and sticky surface (Group B). Group A bacterial cells were characterized as Gram-positive and catalase-negative cocci that grew in pairs or short chains. These were typical features of LAB [26]. On the other hand, Group B cells were Gram-positive and catalase-positive rods forming long chains. These characteristics are not related to LAB but to strains belonging to the genera Bacillus, Corynebacterium, and Listeria [27].

Successful identification (99–100%) was achieved for all the bacterial isolates from each condition by applying PCR analysis and comparing their 16S rRNA sequences with those reported in the EzBioCloud database. The results are presented in Table 2. Two dominant bacterial species of different genotype were identified in all of the examined conditions, namely *Enterococcus casseliflavus* (Group A) and *Bacillus amyloliquefaciens* subsp. *plantarum* (Group B). According to the electrophoresis profiles, the V3 region of 16S rRNA gene had a molecular size of 300 bp. The fact that the collected streams are strongly alkaline (pH value 11.5) due to the debittering of the olive fruit and this condition is not favorable for the survival of the majority of LAB could have influenced the lack of bacterial diversity cultivated on the MRS agar.

*E. casseliflavus*, formerly belonging to the species *Enterococcus flavescens* [28,29], has the ability to survive the debittering stage of the olive fruit and therefore is considered the predominant LAB in the olive flesh during the first 2–3 weeks of lactic acid fermentation. This microorganism has been detected in the flesh of cv. Bella di Cerignola olives after the debittering and during the fermentation stage [16] as well as in the brine during the fermentation of cv. Manzanilla [11] and cv. Sicilian green olives [30]. More importantly, regarding vancomycin resistance, unlike *Enterococcus faecalis* and *Enterococcus faecium*, *E. casseliflavus* is of the VanC phenotype, which demonstrates an inherent low-level resistance to vancomycin [31].

*B. amyloliquefaciens* subsp. *plantarum* belongs to the group of rhizobacteria and grows in the plant roots. The species is commercially used as biofertilizer due to its antagonistic activity against phytopathogens through the enzymatic synthesis of secondary metabolites with antibacterial properties, antifungal properties, and/or the ability to activate metabolic pathways of plant protection [32]. Their mechanism of action is mainly related to the destabilization and alteration of the membrane permeability, leading to cell lysis [33]. *B. amyloliquefaciens* has been molecularly identified in the olive tree and characterized as the species with the strongest antagonistic activity against the main pathogen of olive, the fungus *Verticillium dahlia* [34]. Recently, according to extended phylogenomic analysis, *B. amyloliquefaciens* subsp. *plantarum* was shown as a later heterotypic synonym of *Bacillus velezensis* [35].

The above bacterial isolates were identified for the first time in the processing line of cv. Chalkidiki green table olives.

### 3.3. Technological Characteristics of Enterococcus casseliflavus Isolate

*E. casseliflavus* was designated as the only cultivable indigenous LAB present in the GOW. The ability of the molecularly identified strain to survive the alkaline treatment supports further investigation of its potential to serve as GOW bioaugmentation agent and/or an indigenous starter culture for Spanish-style cv. Chalkidiki green table olive production. The strain used in the further studies was isolated at initial pH value of 11.5 and room temperature conditions, which corresponded to the natural habitat of the species.

#### 3.3.1. Bioaugmentation Ability in Spanish-Style Green Olive Processing Wastewaters

When the *E. casseliflavus* isolate was inoculated in GOW, its growth was indicated by an increase in OD_630_ value from 0.122 ± 0.005 to 1.286 ± 0.008 after 144 h of incubation. The growth coincided with a total depletion of sugars, starting from 2.5 g/L of glucose and 2.6 g/L of fructose. In non-inoculated control, no growth was observed. Thus, further examination in the bioaugmentation of phenolic compounds in GOW was challenging. The data are detailed below.

A total polar phenol content reduction of 73% ± 1% (from 644.9 mg/L ± 16.7 mg/L to 175.2 mg/L ± 9.6 mg/L) at the end of incubation was estimated for the inoculated stream, whereas that for the non-inoculated stream was slightly reduced (<3% reduction). HPLC analysis at 280 nm at key time intervals gave a new insight into the interpretation of the results (Figure S2). Notably, the polar fraction of the fresh GOW polar extracts consisted mainly of simple phenols. As expected, hydroxytyrosol was the main phenolic compound of the stream, reaching 280.5 mg/L ± 10.9 mg/L. Methoxy derivative of hydroxytyrosol (OMeHTyr) and tyrosol were also detected in the fresh GOW at 128.9 mg/L ± 1.6 mg/L and 71.1 mg/L ± 2.0 mg/L, respectively. On the other hand, the phenolic acids and flavonoids, namely, caffeic acid, luteolin-7-*O*-glucoside, and *p*-coumaric acid were at quite low levels (5.0 mg/L ± 0.1 mg/L, 11.7 mg/L ± 0.1 mg/L and 15.0 mg/L ± 0.3 mg/L, respectively). With regard to the phenolic profiles of the treated stream (Figure S2), it is evident that the bacterial strain was able to completely degrade hydroxytyrosol, OMeHTyr, caffeic acid, and *p*-coumaric acid (100% reduction). The simple structure and the high availability of the compounds in the wastewater obviously favored their catabolism by *E. casseliflavus* in the inoculated GOW. In addition, tyrosol and luteolin-7-*O*-glucoside were degraded by 56% ± 1% and 60% ± 1%, respectively, but not completely. This must be related to the lower strain selectivity in the catabolism of the latter compounds. The overall process efficiency was reflected to a COD reduction of 72% (from 30.3 g/L ± 0.1 g/L to 8.6 g/L ± 0.3 g/L).

The above results go beyond previous reports, showing the appropriateness of *E. casseliflavus* for the rapid degradation of hydroxytyrosol and other simple phenols present in the GOW. This is strengthened by the fact that the bioaugmentation ability of the indigenous LAB strain was comparable with that for the exogenous fungus *A. niger*, marked for its effectiveness in our previous work [3]. This finding supports the aim of the current study toward the exploitation of indigenous microbial communities as bioaugmentation agents in GOW.

#### 3.3.2. Resistance to pH, Salinity and Temperature

The resistance of the *E. casseliflavus* isolate to high and low pH values as well as different salt concentrations and temperature, as critical environmental factors related to Spanish-style cv. Chalkidiki green olive fermentation, was modeled. An unblocked central composite design was used to select the best combination of the initial pH value (*X*_1_), NaCl content (*X*_2_, % *w*/*v*), and temperature (*X*_3_, °C) that provide the maximum response value OD_630_ (*Y*). The experimental data for the response variable *Y* at the designed points are shown in Table 1. By applying multiple regression analysis on data, the second-order polynomial equation, in terms of uncoded units, was fitted for the response (*Y*) and simplified in the form shown below (Equation (2)):

(2) $$Y=-2.292+0.3332X_{1}+0.0488X_{2}+0.0776X_{3}+0.00376X_{2}^{2}-0.001240X_{3}^{2}-0.02195X_{1}X_{2}$$

The full second-order model was simplified by omitting the non-significant terms, $X_{1}^{2}$, *X*_1_*X*_3_, and *X*_2_*X*_3_ (*p* > 0.05). Although *X*_3_ had non-significant linear effect on *Y*, the factor was included in Equation (2) as its quadratic effect was significant at the 5% level. Analysis of variance for the model revealed non-significant lack-of-fit (*p* = 0.142). Furthermore, the adjusted and predicted coefficients of determination ($R_{adj}^{2}$0.947 and $R_{pred}^{2}$ 0.832, respectively) were high and close to each other. This finding indicates that the model can be used satisfactorily as a tool to evaluate the effects of the three factors on the bacterial growth and to predict the response for new observations.

**Figure 2:**
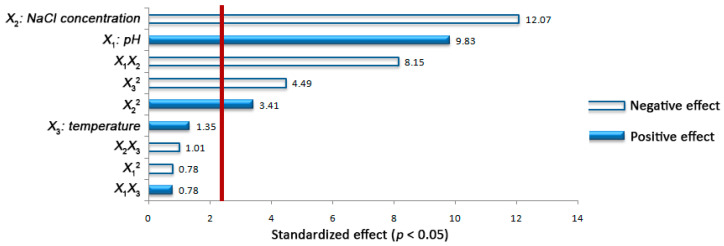
Standardized (*p* < 0.05) Pareto chart in the central composite design for the effects of pH value (*X*_1_), NaCl content (*X*_2_), and temperature (*X*_3_) as well as of their interactions on the growth of the *Enterococcus casseliflavus* isolate (*Y*) in MRS broth. The bars crossing the reference line (red bar) correspond to statistically significant effects (*p* < 0.05).

Examination of Pareto chart (Figure 2) allowed the identification of the main and interaction effects of the independent factors on the response variable. Specifically, the linear term of NaCl content (*X*_2_) had the strongest and negative effect on the bacterial growth. Its quadratic effect was significant and positive on *Y*, signifying that the negative impact of *X*_2_ decelerated beyond its middle level (10% *w*/*v*). What is more, the linear influence of the initial pH value (*X*_1_) on the growth of *E. casseliflavus* was significant and positive, while the quadratic effect of the factor was negative but non-significant on the response. This indicates that a plateau is reached when *X*_1_ approaches its set maximum value (12.4). Although temperature (*X*_3_) appeared to have positive but non-significant linear effect on *Y*, its quadratic term possessed significant and negative effect on the response. The latter denotes a deceleration of the factor impact at values higher than the middle level (30 °C). Additionally, significant and negative interactions were assigned to the examined factors *X*_1_ and *X*_2_ for the bacterial growth, demonstrating that the positive influence of the initial pH value diminishes with the increase of the NaCl content. Finally, non-significant interactions were also recorded for $X_{1}^{2}$, *X*_1_*X*_3_, and *X*_2_*X*_3_ on the response.

**Figure 3:**
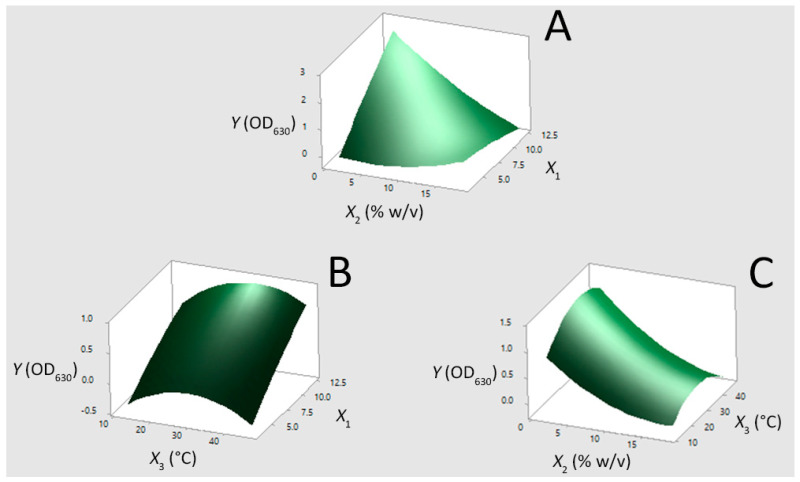
Three dimensional surface plots for the growth of the *Enterococcus casseliflavus* isolate (*Y*) in MRS broth as a function of (**A**) pH value (*X*_1_) and NaCl content (*X*_2_), (**B**) *X*_1_ and temperature (*X*_3_), and (**C**) *X*_2_ and *X*_3_, by keeping the third factor constant at its middle level.

To visualize better the relationship between *Y* and independent factors as well as to assess the optimum fermentation conditions for the maximum value of OD_630_, the fitted polyonomial equation (Equation (2)) was expressed as three-dimensional response surface plots (Figure 3). Figure 3A,B highlights that by increasing the initial pH value from 3.1 up to 12.4, the growth ability of *E. casseliflavus* exponentially increased. This can be due to the fact that the bacterium was able to survive during the debittering stage of the olive fruit by developing homeostatic control mechanisms and/or gene regulatory features at the cellular level [23,24], and therefore the increase of the initial pH value favored the growth media to resemble the natural habitat of the strain. As has already been discussed in our previous study [36], the pH value of the brine during the first days of fermentation in the PDO “Prasines Elies Chalkidikis” production line is high due to the debittering with dilute solution of NaOH. Owing to the ability of the alkali-tolerant *E. casseliflavus* to grow at pH values up to 12.4, this starter culture is expected to dominate the natural microbiota, initiating the lactic acid fermentation from the early phase of the process. This favors a quicker brine acidification, which has been marked as a decisive correction action in the PDO “Prasines Elies Chalkidikis” production line [36], as well as the activity of the lactobacilli in the acidic environment [11].

On the other hand, Figure 3A,C reflects the negative influence of salinity on the bacterial growth. Specifically, the response values decreased as the NaCl content increased up to 10% (*w*/*v*), while a plateau is reached at higher factor values (between 10% *w*/*v* and 18% *w*/*v*). This trend is attributed to the salt stress, which causes the repression of significant cellular functions by altering gene regulation [37,38]. However, considering that the typical range of NaCl content in fermentation brine of Spanish-style cv. Chalkidiki green table olives processing varies between 8% (*w*/*v*) and 9% (*w*/*v*) [36], the ability of the strain to survive at these levels seems promising for its utilization in table olive production. According to Figure 3B,C, it can be deduced that the growth of *E. casseliflavus* was more pronounced at levels of temperature between 30 °C and 37 °C. As the temperature rises beyond the optimum level, the rate of cell denaturation increased [20] and consequently the response values decreased. In addition, the substrate affinity of the bacterial cells decreased at low temperatures due to the stiffening of membrane lipids, which leads to the decrease of membrane permeability [20]. In any case, the molecularly identified *E. casseliflavus* isolate was able to grow at the tested temperature range, i.e., between 13 °C and 47 °C. The latter feature of the strain is quite important since the average fermentation temperature in the PDO “Prasines Elies Chalkidikis” production line is 17 °C [36].

As the model for Equation (2) offers a good estimate to the experimental conditions used to predict the optimum values of the variables. The respective initial pH value, NaCl content, and temperature combinations of 12.4, 1.6% (*w*/*v*), and 36 °C can be recommended as optimum for *Y*. The predicted value for *Y* at the optimum conditions (*Y* = 2.662) fits well with the experimental one (*Y* = 2.303 ± 0.024) since the latter falls within the range of the 95% of confidence (2.298–3.026) and prediction intervals (2.230–3.095).

#### 3.3.3. Resistance to Oleuropein

The ability of the isolated strain *E. casseliflavus* to hydrolyze oleuropein was tested to support further the selection of the isolate as starter culture in table olive production line, aiming to promote the natural debittering of the olive fruit. The available research is focused on the investigation of oleuropein hydrolysis by the enzyme system of *Lactobacillus* spp., e.g., [18,39], while relevant studies for *Enterococcus* spp. are limited [40].

In the experimental tests, the polar extract of the fresh cv. Chalkidiki olive fruit was used as a source of oleuropein. The total polar phenol content in the extract of the olive fruit was determined to be 5215.9 mg/L ± 159.7 mg/L. Based on the RP-HPLC profile at 280 nm (Figure S3), the major phenolic components of the polar extract were oleuropein (4005.1 mg/L ± 61.4 mg/L), and hydroxytyrosol (414.7 mg/L ± 4.6 mg/L), followed by tyrosol (115.2 mg/L ± 1.7 mg/L), decarboxymethyl oleuropein aglycon (103.0 mg/L ± 1.1 mg/L), luteolin (77.8 mg/L ± 1.0 mg/L), and luteolin-7-*O*-glucoside (53.2 mg/L ± 0.6 mg/L).

Table 3 shows the bacterial population as well as the residual content and degradation percentage of the oleuropein after exposure of the strain on a substrate, containing the polar extract of the olive fruit (without or after appropriate dilutions) and bacterial growth factors. Results indicate that the MIC value of oleuropein against the growth of *E. casseliflavus* was 0.9 g/L. Upon exposure of cells to oleuropein concentration of 0.8 g/L, the strain retained approximately 25% of its initial population (visible microbial growth), reaching 1.6 log CFU/mL. The maximum population (4.6 log CFU/mL) was recorded for a substrate with 0.7 g/L of oleuropein. Further decrease in oleuropein concentration (from 0.7 g/L to 0.08 g/L) did not favor bacterial growth due to limited carbon availability. The difficulty of the strain survival at oleuropein levels above 0.8 g/L is also related to the synergistic antimicrobial activity between oleuropein and the other phenolic components of the extract (e.g., hydroxytyrosol, tyrosol). The literature data point out that the MIC of oleuropein for the growth of *E. faecalis* UUMF-EF01 was 3-fold lower (0.1 g/L) in a polar extract of olive leaves containing oleuropein (94%) and a low amount of other phenolic compounds (6%) than in MRS agar (0.3 g/L) [41].

Hydroxytyrosol was formed from oleuropein via the bacterial enzyme activity (mainly *β*-glucosidase and esterase) [40]. In the substrate with 0.8 g/L oleuropein, its content was reduced by 43% (0.5 g/L) with the simultaneous increase of hydroxytyrosol content from 82.8 mg/L ± 1.7 mg/L to 292.4 mg/L ± 7.2 mg/L (Figure S3). The highest efficiency for oleuropein degradation was observed in the substrates with ≤0.2 g/L oleuropein. Under these conditions, hydroxytyrosol was also depleted after 60 h, possibly due to the carbon source limitation. The fact that the concentration of oleuropein remained constant (degradation < 2.6%) in the control substrates confirms that the above changes were attributable to the bacterial activity. According to the literature, the efficiency of LAB for oleuropein degradation varies significantly between the microbial strains. For the same initial concentration of oleuropein (0.8 g/L), the degradation of the compound by *E. casseliflavus* in this study (43%) was found to be 5-fold higher than the corresponding value recorded by *E. faecium* 32 (5–9%) [40]. Additionally, among 105 strains isolated from olive fermentation brine, most of which belonging to *Lactobacillus plantarum* and *Lactobacillus pentosus* species, only the 5 were able to hydrolyze 5 g/L of oleuropein at satisfactory levels (60–90%) [42]. Furthermore, *E. casseliflavus* is expected to effectively grow in olive fermentation brine with oleuropein concentrations 4-fold higher than 0.8 g/L [43]. This hypothesis is supported by the fact that the olive nutrients are diffused to the brine and catabolized to enable microbial growth, while proteins and amino acids can bind with oleuropein and reduce its antimicrobial activity [44]. The adequacy of the strain as a starter culture is also strengthened by the fact that the oleuropein content in the brine is expected to be low (0.2 g/L to 0.3 g/L) in the early stages of fermentation [45,46].

### 3.4. Changes in Hydroxytyrosol during Spontaneous Fermentation of Spanish-Style Green Olive Processing Wastewaters

Oleuropein, the main phenolic component of fresh olive fruit, yields elenolic acid glucoside, and hydroxytyrosol during the debittering stage of Spanish-style green olive processing. The latter compound is a high-value chemical with antibacterial and health beneficial properties such as antioxidant, anti-inflammatory and neuroprotective activities [2]. Toward the recovery of hydroxytyrosol from GOW, its fate was assessed during the spontaneous fermentation of the stream under different temperature and initial pH conditions (Table 4).

In all treatments, in which the initial pH of GOW was uncorrected (11.5), hydroxytyrosol content gradually decreased until complete disappearance (95–100% reduction) after 60 d of fermentation at room temperature and 360 d at the other tested temperatures (15 °C, 30 °C and 50 °C). The above observations are related to the fact that *o*-diphenols are unstable in alkaline environments, resulting in their oxidative degradation by NaOH [47].

**Figure 4:**
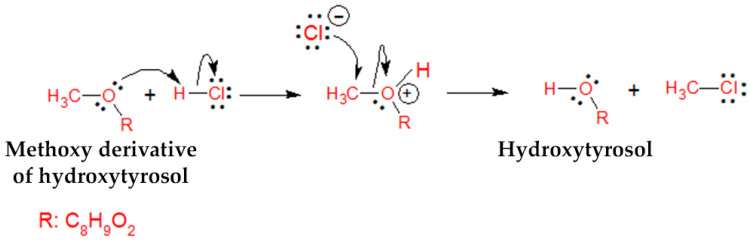
Proposed S_N_2 reaction mechanism between the methoxy derivative of hydroxytyrosol and hydrochloric acid for the formation of hydroxytyrosol.

Adjustment of the initial pH value of GOW to either 3.5 or 5.0 contributed to the increase in hydroxytyrosol content (1.2- to 1.5-fold) from the first day of fermentation followed by the disappearance of OMeHTyr. The phenomenon was attributed to the nucleophilic substitution reaction (S_N_2) of the methoxy group with the HCl used for the acidification of the GOW (Figure 4) [48].

Table 4 designates that there was further increase in the content of hydroxytyrosol (between 444 mg/L and 553 mg/L) in the acidified GOW up to 120 d at all temperatures except for 50 °C, at which thermal decomposition of the compound may have taken place [49]. A main factor of hydroxytyrosol increase was attributed to the hydrolysis of complex phenolic compounds by the indigenous LAB through the action of one or more enzymes. Similar conclusions were drawn by Feki et al. [50], who observed a 4-fold increase in hydroxytyrosol levels after spontaneous fermentation of olive mill wastewaters at 25 °C for 120–150 d. Longer fermentation time (180–360 d) led to a reduction of hydroxytyrosol by 40–70%. The compound degradation by the LAB can be attributed to the conversion of the phenolic alcohol to the corresponding aldehyde derivative via the action of benzyl alcohol dehydrogenase [51].

The above results offer a solid basis to develop an effective biorefinery scheme for the simultaneous LAB isolation and hydroxytyrosol recovery from the GOW. The optimum process scenario proposed in the current study is the initial pH adjustment of GOW to 5.0 and the further spontaneous fermentation of the stream at 30 °C for four months. Fermented GOW offers bioaugmentation agents/starter cultures and the high-value compound, hydroxytyrosol, in a cost-effective way.

## 4. Conclusions

*E. casseliflavus* and *B. amyloliquefaciens* subsp. *plantarum* were isolated and identified as the dominant indigenous bacteria during spontaneous fermentation of GOW. *E. casseliflavus* was found to be an effective bioaugmentation agent. Results also provided evidence of its tolerance to alkalinity and salinity as well as its ability to hydrolyze oleuropein. Hydroxytyrosol formation during GOW fermentation supports further the valorization strategy. Future research should be devoted to the application of *E. casseliflavus* for the simultaneous detoxification and valorization of table olive wastewaters and as a starter culture for table olives (cv. Chalkidiki) production and PDO specification. Additionally, *B. amyloliquefaciens* could be used as a biofertilizer to protect the olive tree from phytopathogens. These innovative approaches are expected to boost a sustainable bioeconomy as well as competitiveness at local and regional levels.

## Figures and Tables

**Table 1:** Experimental design for three-factor five-level central composite design as well as comparison between experimental and predicted response values for the growth of the indigenous isolate *Enterococcus casseliflavus* (*Y*, optical density at 630 nm) in MRS broth.

Run	Factor			Response (Experimental ^1^/Predicted)
	*X*_1_	*X*_2_	*X*_3_	*Y*
1	7.8	10.0	47	0.175/0.228
2	10.5	5.0	40	1.582/1.490
3	7.8	10.0	30	0.481/0.514
4	5.0	15.0	20	0.107/0.172
5	10.5	5.0	20	1.306/1.281
6	7.8	10.0	30	0.565/0.514
7	7.8	10.0	30	0.579/0.514
8	7.8	1.6	30	1.370/1.356
9	5.0	15.0	40	0.119/0.116
10	7.8	18.4	30	0.152/0.205
11	3.1	10.0	30	0.105/−0.015
12	7.8	10.0	13	0.113/0.099
13	7.8	10.0	30	0.459/0.514
14	5.0	5.0	20	0.125/0.178
15	5.0	5.0	40	0.206/0.271
16	10.5	15.0	40	0.208/0.128
17	7.8	10.0	30	0.599/0.514
18	10.5	15.0	20	0.161/0.068
19	12.4	10.0	30	0.764/0.923
20	7.8	10.0	30	0.410/0.514

^1^ Mean value of three independent measurements (*n* = 3).

**Table 2:** Phenotypic characterization and molecular identification of the isolated strains from fermented Spanish-style green olive processing wastewaters under different temperature and initial pH conditions.

Fermentation Conditions		Number of Isolates (Group)	Phenotypic Characterization			Molecular Identification (16S rRNA, Database: EzBioCloud)	
Temperature	Initial pH Value		Cell Morphology	Gram Stain Test	Catalase Test	Reference Strain	Similarity
15 °C	3.5	6 (A)	Cocci	+	–	*Enterococcus casseliflavus* ATCC 49996	99%
		3 (B)	Rods	+	+	*Bacillus amyloliquefaciens* subsp. *plantarum* FZB42	100%
	5.0	6 (A)	Cocci	+	–	*Enterococcus casseliflavus* ATCC 49996	99%
		4 (B)	Rods	+	+	*Bacillus amyloliquefaciens* subsp. *plantarum* FZB42	99%
	11.5	6 (A)	Cocci	+	–	*Enterococcus casseliflavus* ATCC 49996	99%
		2 (B)	Rods	+	+	*Bacillus amyloliquefaciens* subsp. *plantarum* FZB42	100%
30 °C	3.5	5 (A)	Cocci	+	–	*Enterococcus casseliflavus* ATCC 49996	99%
		3 (B)	Rods	+	+	*Bacillus amyloliquefaciens* subsp. *plantarum* FZB42	100%
	5.0	6 (A)	Cocci	+	–	*Enterococcus casseliflavus* ATCC 49996	99%
		4 (B)	Rods	+	+	*Bacillus amyloliquefaciens* subsp. *plantarum* FZB42	100%
	11.5	6 (A)	Cocci	+	–	*Enterococcus casseliflavus* ATCC 49996	100%
		2 (B)	Rods	+	+	*Bacillus amyloliquefaciens* subsp. *plantarum* FZB42	100%
50 °C	3.5	6 (A)	Cocci	+	–	*Enterococcus casseliflavus* ATCC 49996	99%
		3 (B)	Rods	+	+	*Bacillus amyloliquefaciens* subsp. *plantarum* FZB42	100%
	5.0	6 (A)	Cocci	+	–	*Enterococcus casseliflavus* ATCC 49996	100%
		2 (B)	Rods	+	+	*Bacillus amyloliquefaciens* subsp. *plantarum* FZB42	100%
	11.5	7 (A)	Cocci	+	–	*Enterococcus casseliflavus* ATCC 49996	99%
		3 (B)	Rods	+	+	*Bacillus amyloliquefaciens* subsp. *plantarum* FZB42	99%
Room temperature	3.5	6 (A)	Cocci	+	–	*Enterococcus casseliflavus* ATCC 49996	99%
		3 (B)	Rods	+	+	*Bacillus amyloliquefaciens* subsp. *plantarum* FZB42	99%
	5.0	6 (A)	Cocci	+	–	*Enterococcus casseliflavus* ATCC 49996	99%
		4 (B)	Rods	+	+	*Bacillus amyloliquefaciens* subsp. *plantarum* FZB42	99%
	11.5	7 (A)	Cocci	+	–	*Enterococcus casseliflavus* ATCC 49996	100%
		3 (B)	Rods	+	+	*Bacillus amyloliquefaciens* subsp. *plantarum* FZB42	100%

**Table 3:** Bacterial population and oleuropein degradation percentage after exposure of the *Enterococcus casseliflavus* isolate on a substrate, containing the polar extract of the olive fruit (without or after appropriate dilutions) and bacterial growth factors (fermentation time: 3 d).

Initial Oleuropein Content (mg/L)	Bacterial Population (log CFU/mL)	Residual Content of Oleuropein (mg/L)		Oleuropein Degradation (%)	
		*E. casseliflavus*	Control	*E. casseliflavus*	Control
4005.1 ± 61.4	0.0 ± 0.0 ^a^	3923.8 ± 69.4 ^a^	3945.3 ± 75.2 ^a^	2.0	1.5
2093.5 ± 30.3	0.0 ± 0.0 ^a^	2051.9 ± 34.9 ^b^	2054.9 ± 24.0 ^b^	2.0	1.8
898.2 ± 12.9	0.0 ± 0.0 ^a^	884.0 ± 12.5 ^c^	878.0 ± 15.7 ^c^	1.6	2.3
806.7 ± 14.9	1.6 ± 0.1 ^b^	461.9 ± 13.3 ^d^	785.5 ± 19.1 ^d^	42.7	2.6
715.8 ± 10.3	4.6 ± 0.1 ^c^	303.5 ± 8.5 ^e^	704.9 ± 18.0 ^e^	57.6	1.5
603.3 ± 13.1	4.0 ± 0.1 ^d^	156.8 ± 5.1 ^f^	590.8 ± 10.9 ^f^	74.0	2.1
396.7 ± 8.1	3.4 ± 0.1 ^e^	50.9 ± 1.4 ^g^	391.4 ± 9.4 ^g^	87.2	1.3
198.3 ± 3.5	3.0 ± 0.1 ^f^	0.0 ± 0.0 ^h^	194.9 ± 4.3 ^h^	100.0	1.7
83.4 ± 1.2	2.7 ± 0.1 ^g^	0.0 ± 0.0 ^h^	81.6 ± 1.2 ^i^	100.0	2.2

Data are the mean ± standard deviation (*n* = 3). Different lowercase letters in the same column represent significant differences in values (*p* < 0.05). Control: Non-inoculated substrate.

**Table 4:** Changes in hydroxytyrosol content during the spontaneous fermentation of Spanish-style green olive processing wastewaters under different temperature and initial pH conditions.

Fermentation Conditions		Hydroxytyrosol Content (mg/L) ^1^								
Temperature	Initial pH Value	Fermentation Time (d)								
		1	2	10	20	30	60	120	180	360
15 °C	3.5	420.9 ± 26.7 ^a,A^	371.6 ± 16.8 ^b,A^	349.3 ± 15.4 ^bc,A^	328.0 ± 15.9 ^c,AF^	326.8 ± 15.4 ^c,A^	474.7 ± 24.5 ^d,A^	490.5 ± 16.8 ^d,A^	195.9 ± 11.3 ^e,A^	72.6 ± 5.1 ^f,A^
	5.0	417.3 ± 17.5 ^a,AD^	356.6 ± 13.6 ^b,AF^	359.3 ± 12.5 ^b,A^	418.2 ± 18.8 ^a,B^	328.0 ± 16.1 ^c,A^	332.5 ± 11.5 ^bc,B^	501.2 ± 19.2 ^d,AH^	273.0 ± 12.6 ^e,B^	120.4 ± 9.2 ^f,B^
	11.5	234.0 ± 10.0 ^a,B^	135.6 ± 5.4 ^bc,B^	133.6 ± 9.5 ^b,B^	150.4 ± 8.2 ^bc,C^	152.9 ± 8.2 ^c,B^	238.0 ± 17.0 ^a,C^	222.5 ± 14.1 ^a,B^	35.6 ± 4.5 ^d,C^	2.2 ± 0.1 ^e,C^
30 °C	3.5	336.7 ± 13.5 ^a,C^	324.6 ± 13.0 ^a,C^	363.7 ± 19.7 ^b,A^	319.5 ± 13.5 ^a,A^	291.9 ± 9.9 ^c,C^	361.9 ± 15.4 ^b,D^	443.7 ± 17.4 ^d,C^	258.6 ± 12.7 ^e,B^	59.7 ± 2.1 ^f,D^
	5.0	392.8 ± 15.1 ^ad,D^	332.5 ± 13.9 ^b,C^	431.5 ± 15.1 ^c,C^	433.1 ± 16.9 ^c,B^	377.1 ± 14.7 ^a,D^	409.6 ± 16.9 ^cd,E^	552.9 ± 26.9 ^e,D^	240.1 ± 19.3 ^f,D^	112.8 ± 7.7 ^g,E^
	11.5	231.0 ± 14.1 ^a,B^	210.1 ± 10.2 ^b,D^	207.2 ± 8.6 ^b,D^	254.0 ± 13.3 ^c,D^	259.9 ± 15.6 ^c,E^	266.8 ± 13.3 ^c,F^	206.8 ± 12.9 ^b,B^	45.5 ± 5.9 ^d,C^	13.2 ± 1.2 ^e,F^
50 °C	3.5	343.0 ± 16.1 ^a,C^	295.0 ± 15.4 ^b,E^	324.9 ± 12.9 ^a,E^	295.2 ± 13.8 ^b,E^	295.3 ± 12.2 ^b,C^	302.0 ± 14.0 ^b,G^	343.1 ± 16.1 ^a,E^	144.5 ± 7.0 ^c,E^	54.3 ± 1.7 ^d,D^
	5.0	339.8 ± 16.7 ^a,C^	335.6 ± 14.5 ^a,CF^	308.0 ± 13.9 ^b,E^	346.2 ± 13.1 ^a,FH^	220.7 ± 10.8 ^c,F^	231.5 ± 11.1 ^c,C^	253.8 ± 8.6 ^d,F^	84.0 ± 5.8 ^e,F^	23.7 ± 0.9 ^f,G^
	11.5	194.1 ± 7.2 ^ab,E^	207.9 ± 7.6 ^a,D^	208.5 ± 9.6 ^a,D^	203.3 ± 9.5 ^ab,G^	189.4 ± 9.4 ^b,G^	133.0 ± 12.0 ^c,H^	139.6 ± 8.9 ^c,G^	7.5 ± 0.7 ^d,G^	0.0 ± 0.0 ^d,C^
Room temperature	3.5	323.4 ± 14.3 ^a,C^	267.9 ± 11.7 ^b,G^	418.5 ± 20.7 ^c,C^	432.7 ± 18.0 ^ce,B^	365.0 ± 16.6 ^d,D^	446.4 ± 15.0 ^e,I^	519.3 ± 16.8 ^f,H^	298.3 ± 14.2 ^a,H^	144.9 ± 6.9 ^g,H^
	5.0	324.9 ± 17.4 ^a,C^	321.8 ± 14.8 ^a,C^	355.7 ± 17.6 ^b,A^	365.4 ± 15.2 ^b,H^	320.9 ± 11.7 ^a,A^	382.2 ± 14.3 ^b,D^	456.3 ± 19.4 ^c,C^	233.9 ± 13.9 ^d,D^	107.2 ± 4.2 ^e,E^
	11.5	203.5 ± 7.5 ^a,E^	241.0 ± 15.3 ^b,H^	175.2 ± 10.2 ^c,F^	159.4 ± 12.4 ^d,C^	63.1 ± 4.6 ^e,H^	0.0 ± 0.0 ^f,J^	0.0 ± 0.0 ^f,I^	0.0 ± 0.0 ^f,G^	0.0 ± 0.0 ^f,C^

^1^ Initial hydroxytyrosol content: 280.5 mg/L ± 10.9 mg/L. Data are the mean ± standard deviation (*n* = 3). Different lowercase letters in the same row or capital letters in the same column represent significant differences in values (*p* < 0.05).

## Supplementary Materials

The following are available online at https://www.mdpi.com/2076-2607/8/9/1274/s1; Table S1: Levels of factors in actual and coded values used in the experimental design; Figure S1: Changes in the pH value during the spontaneous fermentation of Spanish-style green olive processing wastewaters with an initial pH value of 3.5, 5.0 or 11.5 at 15 °C, 30 °C, 50 °C, and room temperature; Figure S2: RP-HPLC phenolic profiles at 280 nm of Spanish-style green olive processing wastewaters prior (0 d) and after incubation without (control) or with the *Enterococcus casseliflavus* isolate at 37 °C for 7 d under static conditions; Figure S3: RP-HPLC phenolic profiles at 280 nm of a substrate containing a diluted polar extract of the olive fruit with 806.7 mg/L initial oleuropein concentration and bacterial growth factors prior (0 d) and after incubation with the *Enterococcus casseliflavus* isolate at 37 °C for 3 d under static conditions.

## Abbreviations

CFU: colony forming units; COD: chemical oxygen demand; GOW: Spanish-style green olive processing wastewaters; LAB: lactic acid bacteria; MIC: minimum inhibitory concentration; MRS: Man, Rogosa and Sharpe; OD: optical density; OMeHTyr: methoxy derivative of hydroxytyrosol; PCR: polymerase chain reaction; PDO: protected designation of origin; RP-HPLC: reversed-phase high-performance liquid chromatography.
