# Supplementary material for: Processing Wastewaters from Spanish-Style cv. Chalkidiki Green Olives: A Potential Source of Enterococcus casseliflavus and Hydroxytyrosol
Source: Microorganisms. 2020 Aug 21;8(9):1274. doi: 10.3390/microorganisms8091274 (PMC7564576; doi:10.3390/microorganisms8091274)
Supplement: Supplementary file 1 [file microorganisms-08-01274-s001.pdf]

microorganisms

Supplementary materials for the article:

Processing wastewaters from Spanish-style cv. Chalkidiki green olives: A potential source of *Enterococcus casseliflavus* and hydroxytyrosol

Eugenia Papadaki[1], George Botsaris[2], Eleftheria Athanasiadi[1] and Fani Th. Mantzouridou[1,*]

[1] Laboratory of Food Chemistry and Technology, Department of Chemistry, Aristotle University of Thessaloniki, 54124 Thessaloniki, Greece; epapadaki@chem.auth.gr (E.P.); aelefther@chem.auth.gr (E.A.)
[2] Department of Agricultural Sciences, Biotechnology and Food Science, Cyprus University of Technology, 50329 Limassol, Cyprus; george.botsaris@cut.ac.cy
* Correspondence: fmantz@chem.auth.gr; Tel.: +30 2310 997774

This document contains supplementary materials:

Table S1: Levels of factors in actual and coded values used in the experimental design.

Figure S1: Changes in the pH value during the spontaneous fermentation of Spanish-style green olive processing wastewaters with an initial pH value of 3.5, 5.0 or 11.5 at 15 °C (A), 30 °C (B), 50 °C (C), and room temperature (D).

Figure S2: RP-HPLC phenolic profiles at 280 nm of Spanish-style green olive processing wastewaters prior (0 d) and after incubation without (control) or with the *Enterococcus casseliflavus* isolate at 37 °C for 7 d under static conditions.

Figure S3: RP-HPLC phenolic profiles at 280 nm of a substrate containing a diluted polar extract of the olive fruit with 806.7 mg/L initial oleuropein concentration and bacterial growth factors prior (0 d) and after incubation with the *Enterococcus casseliflavus* isolate at 37 °C for 3 d under static conditions.

Table S1. Levels of factors in actual and coded values used in the experimental design.

Factor	Variable	Levels				
		Coded values[1]				
		$-\alpha$	-1	0	+1	$+\alpha$
		Actual values				
Initial pH value	X_1	3.1	5.0	7.8	10.5	12.4
NaCl content (%, w/v)	X_2	1.6	5.0	10.0	15.0	18.4
Temperature (°C)	X_3	13	20	30	40	47

[1] $Coded\ value = \frac{actual\ \ level - (high\ level + low\ \ level)/2}{(high\ level - low\ \ level)/2}$, where $a = 2^{n/4}$, n = the number of variables and −1, 0, +1 correspond to the low-, mid- and high-level of X_i, respectively.

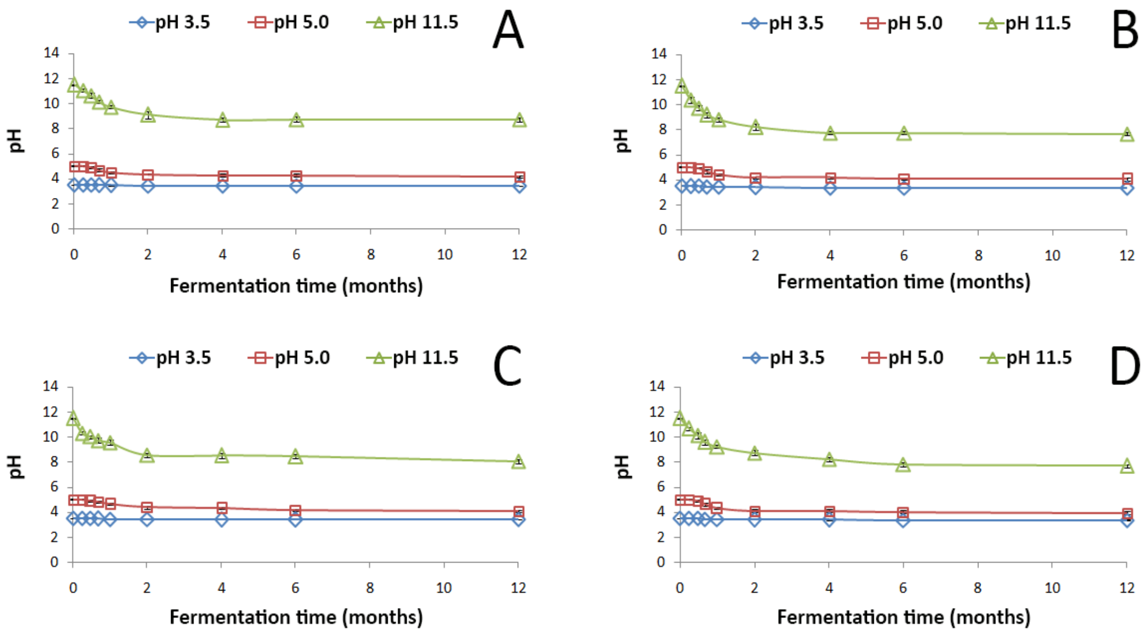

Figure S1. Changes in the pH value during the spontaneous fermentation of Spanish-style green olive processing wastewaters with an initial pH value of 3.5, 5.0 or 11.5 at 15 °C (A), 30 °C (B), 50 °C (C), and room temperature (D). Data points are mean values of 5 independent experiments × 3 measurements (n = 15) and error bars represent the standard deviation of the mean value.

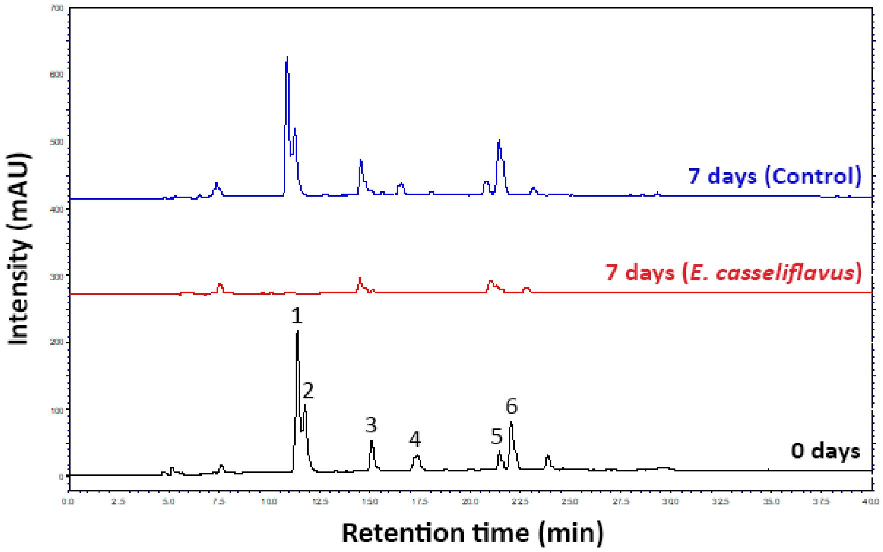

Figure S2. RP-HPLC phenolic profiles at 280 nm of Spanish-style green olive processing wastewaters prior (0 d) and after incubation without (control) or with the *Enterococcus casseliflavus* isolate at 37 °C for 7 d under static conditions. Peaks: (1) Hydroxytyrosol, (2) Methoxy derivative of hydroxytyrosol, (3) Tyrosol, (4) Caffeic acid, (5) Luteolin-7-*O*-glucoside, (6) *p*-Coumaric acid.

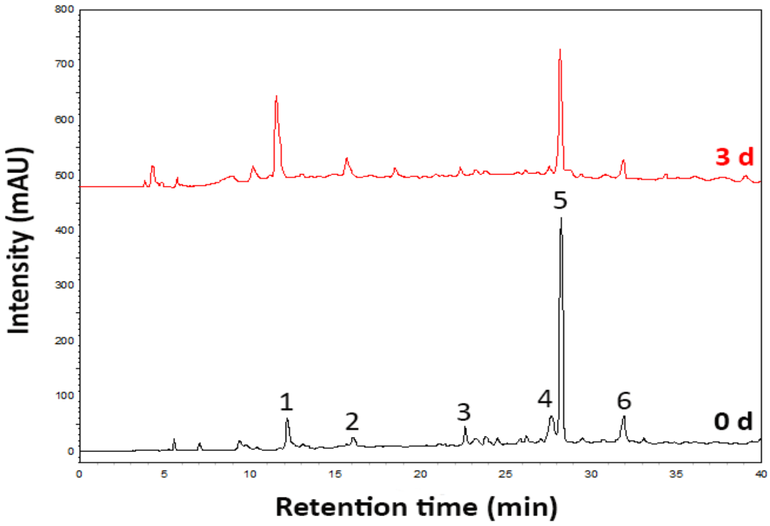

Figure S3. RP-HPLC phenolic profiles at 280 nm of a substrate containing a diluted polar extract of the olive fruit with 806.7 mg/L initial oleuropein concentration and bacterial growth factors prior (0 d) and after incubation with the *Enterococcus casseliflavus* isolate at 37 °C for 3 d under static conditions. Peaks: (1) Hydroxytyrosol, (2) Tyrosol, (3) Luteolin-7-*O*-glucoside, (4) Decarboxymethyl oleuropein aglycon, (5) Oleuropein, (6) Luteolin.

© 2020 by the authors. Submitted for possible open access publication under the terms and conditions of the Creative Commons Attribution (CC BY) license (http://creativecommons.org/licenses/by/4.0/).
